# Cognition and brain oxygen metabolism improves after bariatric surgery-induced weight loss: A pilot study

**DOI:** 10.3389/fendo.2022.954127

**Published:** 2022-12-09

**Authors:** Nareen Anwar, Wesley J. Tucker, Nancy Puzziferri, T. Jake Samuel, Vlad G. Zaha, Ildiko Lingvay, Jaime Almandoz, Jing Wang, Edward A. Gonzales, Robert Matthew Brothers, Michael D. Nelson, Binu P. Thomas

**Affiliations:** ^1^ Department of Biomedical Engineering, University of Texas at Dallas, Richardson, TX, United States; ^2^ Advanced Imaging Research Center, University of Texas Southwestern Medical Center, Dallas TX, United States; ^3^ Department of Kinesiology, University of Texas at Arlington, Arlington, TX, United States; ^4^ Department of Surgery, Oregon Health and Science University, Portland, OR, United States; ^5^ Department of Internal Medicine, University of Texas Southwestern Medical Center, Dallas, TX, United States; ^6^ Department of Population and Data Sciences, University of Texas Southwestern Medical Center, Dallas, TX, United States; ^7^ Department of Radiology, University of Texas Southwestern Medical Center, Dallas, TX, United States; ^8^ Department of Bioengineering, University of Texas at Arlington, Arlington, TX, United States

**Keywords:** obesity, cognition, cerebral metabolic rate of oxygen, bariatric surgery, sleeve gastrectomy, cerebral blood flow, oxygen extraction fraction, venous oxygenation

## Abstract

**Objective:**

The primary objectives of this pilot study were to assess cognition and cerebral metabolic rate of oxygen (CMRO_2_) consumption in people with severe obesity before (baseline), and again, 2- and 14-weeks after sleeve gastrectomy bariatric surgery.

**Methods:**

Six people with severe/class 3 obesity (52 ± 10 years, five females, body mass index (BMI) = 41.9 ± 3.9 kg/m^2^), and 10 normal weight sex- and age-matched healthy controls (HC) (48 ± 6 years, eight females, 22.8 ± 1.9 kg/m^2^). Global CMRO_2_ was measured non-invasively using MRI and cognition using the Integneuro testing battery.

**Results:**

Following a sleeve gastrectomy induced weight loss of 6.4 ± 2.5 kg (% total-body-weight-lost = 5.4) over two-weeks, cognition total scores improved by 0.8 ± 0.5 T-scores (p=0.03, 15.8% improvement from baseline). Weight loss over 14-weeks post-surgery was 15.4 ± 3.6 kg (% total-body-weight-lost = 13.0%) and cognition improved by 1.1 ± 0.4 (p=0.003, 20.6% improvement from baseline). At 14-weeks, cognition was 6.4 ± 0.7, comparable to 6.0 ± 0.6 observed in the HC group. Baseline CMRO_2_ was significantly higher compared to the HC (230.4 ± 32.9 vs. 177.9 ± 33.9 µmol O_2_/100 g/min, p=0.02). Compared to baseline, CMRO_2_ was 234.3 ± 16.2 µmol O_2_/100 g/min at 2-weeks after surgery (p=0.8, 1.7% higher) and 217.3 ± 50.4 at 14-weeks (p=0.5, 5.7% lower) after surgery. 14-weeks following surgery, CMRO_2_ was similar to HC (p=0.17).

**Conclusion:**

Sleeve gastrectomy induced weight loss was associated with an increase in cognition and a decrease in CMRO_2_ observed 14-weeks after surgery. The association between weight loss, improved cognition and CMRO_2_ decrease should be evaluated in larger future studies.

## Introduction

Obesity is a global epidemic and more than one-third of the world’s population is over-weight, with excess body weight linked to a variety of health concerns (1). In the U.S. in 2020,42.4% of adults had obesity and 9.2% had severe obesity (body mass index [BMI] > 40 kg/m^2^) (2), with annual direct healthcare costs attributed to excess body weight exceeding $480 billion (3, 4). Obesity is associated with cardiometabolic diseases such as type 2 diabetes mellitus, hypertension, hypercholesterolemia, cerebral small vessel disease, and Alzheimer’s disease, all of which lead to accelerated aging of the body and brain (5–8). Obesity has also been linked to decreased cognitive function, especially memory, executive function, processing speed, attention and decision making (9, 10). However, less is known regarding the underpinning mechanisms of the relationship between obesity and cognition.

Brain health can be assessed by measurements of brain vascular dilation, neural energy consumption, and cognitive function. To evaluate the effects of obesity on brain health we previously assessed brain vascular dilation as indexed by cerebral vascular reactivity (CVR), i.e. the vasodilatory responsiveness of the cerebral vasculature during a hypercapnic challenge induced by CO_2_ inhalation, and assessed cognitive function using validated neurocognitive testing (11). We observed blunting of CVR in the middle cerebral artery (MCA) and cognitive function in participants with obesity compared to those of age-matched healthy weight controls (11). This indicates that cerebral vascular dilatory responsiveness is decreased with obesity, which may have partly contributed to the diminished cognitive function (9–11).

Measurement of the cerebral metabolic rate of oxygen (CMRO_2_) provides information about the brain’s neural energy consumption (12, 13). CMRO_2_ assessment has provided insight into brain health in mild cognitive impairment (14), multiple sclerosis (15), neonates (16), addiction (17), Alzheimer’s disease (18) and to assess the effect of hyperoxia gas inhalation on the brain (19). However, little is known regarding cerebral oxygen metabolism in people with obesity.

Bariatric surgery is an effective long-term treatment for obesity that leads to improvements in various conditions including hypertension (20), diabetes (21), and neurocognitive function (22). However, other mechanisms with which bariatric surgery improves neurocognitive function remain to be elucidated. Emerging evidence suggests that bariatric surgery modulates a number of molecular targets that may improve vascular function, such as attenuation of oxidative stress and inflammation (23, 24), and improvements in vascular endothelial function (23); each of which could improve cerebral oxygen metabolism.

Based on this background, the present pilot study tested the hypothesis that CMRO_2_ is higher in people with obesity, and improves with sleeve gastrectomy bariatric surgery. We further hypothesized that CMRO_2_ would be associated with cognitive function. To accomplish this goal, we studied participants with severe/class 3 obesity before and after sleeve gastrectomy and compared their CMRO_2_ and cognitive function to that from healthy weight controls with similar age and sex. CMRO_2_ and cognitive function were assessed in people with obesity at 2-weeks and 14-weeks post sleeve gastrectomy to assess the early and medium term changes after surgery induced weight loss.

## Materials and methods

### Study objectives

This was a non-randomized observational pilot study. The primary objective was to assess the changes in CMRO_2_ and cognition in bariatric surgery candidates (BSC) with severe/class 3 obesity pre- and post-surgery at 2- and 14-weeks. All CMRO_2_ and cognitive function measures were compared to healthy normal weight controls of similar age and sex as the BSC group (HC). Measurements on HC were performed at one time. Another group of young (18-39 years) healthy normal weight reference controls (RC) were evaluated cross-sectionally to assess the effect of age (independent of BMI) on CMRO_2_. All participants refrained from alcohol and caffeine for a minimum of 12 h prior to all data collection visits. The study was reviewed and approved by the University of Texas Southwestern Medical Center Institutional Review Board and all participants provided written informed consent prior to participation.

### Participants

BSC with severe/class 3 obesity were recruited from the weight management clinics at the UT Southwestern Health System (Dallas, TX). Healthy control participants (HC and RC) were recruited from the community during the same dates.

### Inclusion criteria

We recruited three separate groups of adults (age>18 years): (1) BSC with BMI 35-40 kg/m^2^ with co-morbidities or BMI >40 kg/m^2^, who planned to undergo bariatric surgery for weight management, (2) HC with BMI 18.5-24.9 kg/m^2^, with similar age and sex as the BSC group, and free of underlying co-morbidities, and (3) RC with BMI 18.5-24.9 kg/m^2^ (18-39 years).

### Exclusion criteria

Bariatric surgery candidates were excluded if they had significant anemia (hemoglobin< 10 mg/dl), abnormal renal function (serum creatinine above normal limit for age and sex), chronic respiratory conditions (chronic obstructive pulmonary disease or asthma), pregnancy, waist circumference > 65 in (1.651 meters), incretin mimetic or dipeptidyl peptidase IV inhibitor use during the prior 3 months.

Control participants (both HC and RC), were excluded for history of cardiovascular (e.g. hypertension, type 2 diabetes) or cerebrovascular diseases (e.g. history of stroke, transient ischemic attack), major psychiatric or neurological disorders. All participants were also excluded if they reported any contraindications to MRI.

### Bariatric surgery

All BSC included in this study underwent sleeve gastrectomy surgery at the UT Southwestern Health System (Dallas, TX).

### Anthropometric assessment

Height and weight were measured for all participants with a standard stadiometer and scale (Health-O-Meter, Sun Beam Inc., Boca Raton, FL, USA) for calculation of BMI in kg/m^2^. Waist circumference measurements were taken at the midpoint between lowest rib & top of hip using a standard Gullick tape measure. All anthropometric measurements were performed in duplicate and averaged with participants wearing only a hospital gown.

### Cognitive function assessment

Cognitive function was assessed in BSC pre- and post-surgery and in HC using Integneuro computerized testing battery (Brain Resources Ltd., Australia). Cognitive function in multiple domains was tested and a composite score was generated from all domains. The assessed domains included response speed, impulsivity, attention, information processing, memory, executive function, and emotion identification. The same cognitive tests were repeated in BSC at 2-weeks and 14-weeks after surgery. In a prior study, carry-over effects from repeated cognitive function testing were not observed when tests were performed more than a week apart (25).

### MRI experiments

Experiments were performed on a 3 Tesla MRI scanner using an 8-channel head coil (Philips Healthcare, Best, The Netherlands) for signal reception. A body coil was used for radio-frequency transmission. Foam padding was placed around the head to minimize motion during MRI scan acquisition. Global CMRO_2_ and the associated oxygen extraction fraction (OEF) and global cerebral blood flow are functional MRI biomarkers that were measured in the brain for all study participants.

### Global CMRO_2_

We used a validated technique to measure the brain’s global oxygen metabolism using magnetic resonance imaging (MRI) (26–28). The technique does not require any exogenous tracer, is acquired on a standard 3 Tesla MRI scanner and has a test retest coefficient of variability of less than 4% (29). This method provides quantitative global brain metabolism results expressed as µmol O_2_/100 g/min brain tissue. CMRO_2_ is calculated using the Fick principle based on the brain’s arterio-venous difference in oxygen content:


(1) $$CMRO_{2}=CBF\times OEF\times Ca=CBF\times (Ya–Yv)\times Ca$$

where CBF is the cerebral blood flow, measured with MRI and represents the amount of blood supply to the brain. Ya is the arterial blood oxygen saturation fraction (in %), measured with a pulse oximeter (*In vivo*, Philips Healthcare, Best, The Netherlands), placed on the index finger. Yv is the venous oxygen saturation fraction (in %), measured with MRI, and OEF = (Ya - Yv) is the OEF. Ca is a constant representing the oxygen carrying capacity of unit volume of blood and is 8.97 µmol O_2_/mL blood (30). Ca is adjusted for hematocrit, and a hematocrit value of 0.42 was chosen for males and 0.4 was chosen for females (14). The scan duration of a complete set of CMRO_2_ measurement is 4 min.

CBF is quantified using the phase contrast (PC) MRI technique. The PC MRI technique is routinely used to quantify CBF and extensively described previously (14, 31). Imaging parameters for the PC scan are as follows: single-slice acquisition, voxel size 0.45 × 0.45 × 5 mm^3^, field-of-view (FOV) = 230 × 230 × 5 mm^3^, maximum velocity encoding = 80 cm/s, and scan duration = 30 s. The flux in the four major feeding arteries, left and right internal carotid arteries and the left and right vertebral arteries is measured using an in-house MATLAB (Math-works, Natick, MA, USA) script using methods extensively described previously (14, 31). Briefly, the combined flux from the above mentioned four major feeding arteries is calculated to determine the total flow as mL/min. To determine CBF in mL/100g/min, total brain volume, which is the sum of gray matter and white matter, is obtained from the high-resolution T_1_-weighted magnetization-prepared-rapid-acquisition-of-gradient-echo image (voxel size = 1 × 1 × 1mm^3^, scan duration = 4 min) using functions from the Functional magnetic resonance imaging of the brain Software Library (FSL, Oxford University, Oxford, UK) and normalized to the CBF.

Yv (oxygenation in venous vessels) was measured using T_2_‐Relaxation‐Under‐Spin‐Tagging (TRUST) MRI (14, 26). Briefly, TRUST is based on T2 relaxation time rather than the MRI signal strength itself. The TRUST sequence used the following imaging parameters: single-shot echo-planar imaging acquisition in the axial plane, voxel size = 3.44 × 3.44 × 5 mm^3^, FOV = 220 × 220 × 5 mm^3^, repetition time (TR) = 3000 ms, echo time (TE) = 3.6 ms, inversion time (TI) = 1022 ms, labeling slab thickness = 80 mm, gap between the imaging slice and labeling slab = 20 mm, and four different T_2_ weightings, with effective TE = 0 ms, 40 ms, 80 ms, and 160 ms, corresponding to 0, 4, 8, and 16 refocusing pulses during the T_2_ preparation in the pulse sequence, and scan duration = 1 min 12s.

### Statistical analysis

Data are presented as mean ± SD. Repeated measures ANOVA was used to assess cognitive function, and CMRO_2_ in BSC before, 2- and 14-weeks after surgery. Unpaired t-tests were used to assess the differences in CMRO_2_ between the group of BSC (three time points) and the HC group. The Pearson correlation coefficient was used to measure the relationship between BMI and cognitive function total score, as well as the relationships between both BMI and waist circumference and CMRO_2_ and CBF. The significance level alpha was 0.05; no corrections for multiple comparisons was performed.

## Results

### Participants

Six BSC with severe/class 3 obesity (52 ± 10 years, five females, 41.9 ± 3.9 kg/m^2^), 10 HC of similar age (48 ± 6 years, 8 females, 22.8 ± 1.9 kg/m^2^), and seven RC (24 ± 5 years, 2 females, 23.1 ± 1.9 kg/m^2^) participated in this study. All participants had at minimum a high school education. Participant characteristics at baseline have previously been published, (11) with pertinent information included in **Table 1**. Prior to surgery, CMRO_2_ was significantly higher in BSC than HC (230.4 ± 32.9 µmol O_2_/100 g/min vs. 177.9 ± 33.9 µmol O_2_/100 g/min, p=0.02), while there was no significant difference in cognitive function (5.3 ± 0.7 vs. 6.0 ± 0.6, p=0.07). Cognitive function was only assessed in five BSC as the neurocognitive software was not available for our first participant. CMRO_2_ data was not acquired for two of 10 participants from the HC group due to technical difficulties.

**Table 1:** Baseline participants’ characteristics by group.

	Young healthy reference control (RC) group	Healthy normal weight control (HC) group	Bariatric surgery candidates (BSC) group	p-value: BSC versus HC
**N**	7	10	6	–
**Sex (M, F)**	(5, 2)	(2, 8)	(1, 5)	–
**Age (years)**	24 ± 5	48 ± 6	52 ± 10	0.36
**Ethnicity**				
** Non-Hispanic**	6 (86%)	9 (90%)	6 (100%)	–
** Hispanic**	1 (14%)	1 (10%)	–	–
**Race**				
** White**	6 (86%)	9 (90%)	4 (67%)	–
** Black**	–	–	1 (17%)	–
** Asian**	1 (14%)	1 (10%)	–	–
** American Indian**	–	–	1 (17%)	–
**Weight (kg)**	70.3 ± 8.6	66.3 ± 7.3	118.1 ± 18.0	<0.001
**Body mass index (kg/m^2^)**	23.1 ± 1.9	22.8 ± 1.9	41.9 ± 3.9	<0.0001
**CMRO_2_ (*μ*mol/100g/min)**	174.0 ± 22.2	177.9 ± 33.9	230.4 ± 32.9	0.02
**Cognitive function total (T-score)**	–	6.0 ± 0.6	5.3 ± 0.7	0.07
**Comorbidities**				
** Hypertension**	–	2 (20%)	5 (83%)	–
** Hypercholesterolemia**	–	–	4 (67%)	–
** Diabetes**	–	–	2 (33%)	–
** Hypothyroidism**	–	2 (20%)	3 (50%)	–
** Anxiety/depression**	–	2 (20%)	4 (67%)	–
**Medications**				
** ACE inhibitor**	–	–	4 (67%)	–
** Beta-blocker**	–	–	2 (33%)	–
** Statin**	–	–	1 (17%)	–
** Biguanide**	–	–	2 (33%)	–
** Levothyroxine**	–	2 (20%)	3 (50%)	–
** SSRIs**	–	2 (20%)	3 (50%)	–

Values are mean ± standard deviation; “-”: test not done or data not available.

### Effect of sleeve gastrectomy bariatric surgery

**Figure 1:**
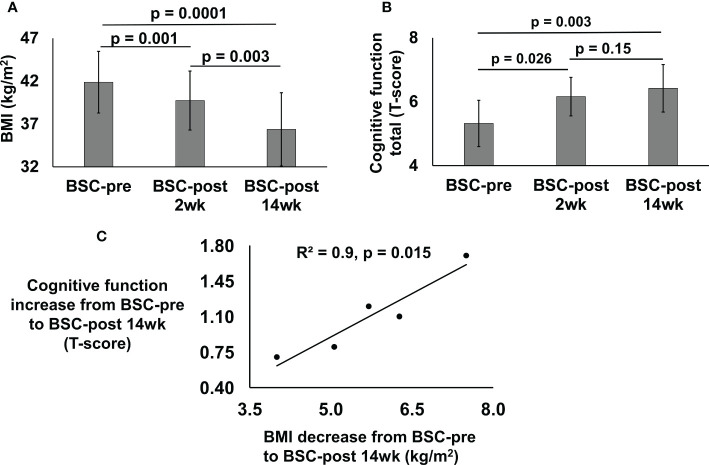
BMI (kg/m2) **(A)** and cognitive function total (T-score) **(B)** of the bariatric surgery candidates (BSC) group with severe/class 3 obesity before (BSC-pre), two weeks after (BSC-post 2wk), and fourteen weeks after (BSC-post 14wk) surgery. BMI data was assessed for 6 participants; cognitive function was assessed for 5 participants. Cross correlation between the BMI decrease from BSC-pre to BSC-post 14wk and cognitive function increase from BSC-pre to BSC-post 14 wk **(C)**, p = 0.015.

Sleeve gastrectomy bariatric surgery induced a weight loss of 6.4 ± 2.5 kg (% total-body-weight-lost = 5.4) over 2-weeks and 15.4 ± 3.6 kg (% total-body-weight-lost = 13.0%) over 14-weeks. Pre-surgery, mean BMI of the BSC group was 41.9 ± 3.6 kg/m^2^, which significantly decreased to 39.7 ± 3.4 kg/m^2^, 2-weeks post-surgery and to 36.4 ± 4.5 kg/m^2^, 14-weeks post-surgery compared to pre-surgery (**Figure 1A**).

Cognitive function improved in the BSC group 2-weeks (6.2 ± 0.6, p=0.03) and 14-weeks post-surgery (6.4 ± 0.7, p=0.003) compared to their cognition pre-surgery (**Figure 1B**). Cognitive function scores improved by 0.8 ± 0.5 T-scores (p=0.03, 15.8% improvement from baseline over 2-weeks) and 1.1 ± 0.4 (p=0.003, 20.6% improvement from baseline over 14-weeks).

Pearson correlation for the change from pre-surgery to 14-weeks post-surgery in cognitive function and BMI was R^2^ = 0.9 (p=0.015) (**Figure 1C**).

**Figure 2:**
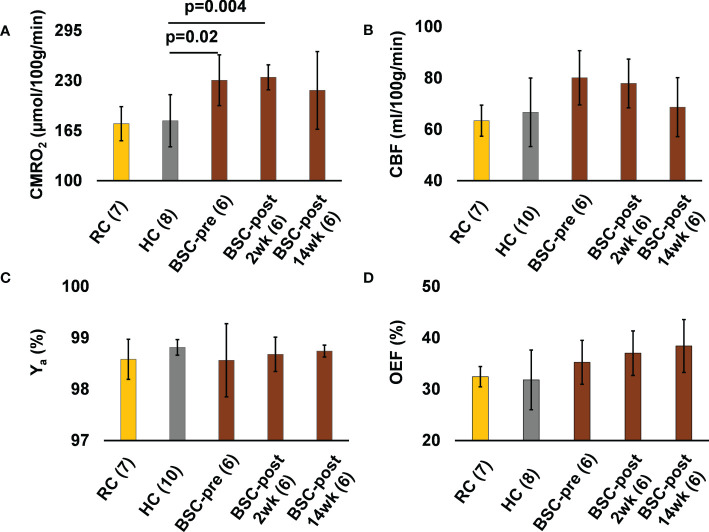
The CMRO2 **(A)**, CBF **(B)**, Ya **(C)**, and OEF **(D)** values of the young healthy weight reference controls (RC) group, healthy normal weight controls (HC) of similar age and sex as the BSC group, and the bariatric surgery candidates group before (BSC-pre), two weeks after (BSC-post 2wk), and fourteen weeks after (BSC-post 14wk) surgery. Numbers in brackets next to each group label indicates the number of participants in the group.

Baseline CMRO_2_ was significantly higher in the BSC group (230.4 ± 32.9 vs. 177.9 ± 33.9 µmol O_2_/100 g/min, p=0.02) compared to the HC group (**Table 2**). Compared to baseline, CMRO_2_ was 234.3 ± 16.2 µmol O_2_/100 g/min at 2-weeks after surgery (p=0.8, 1.7% higher) and 217.3 ± 50.4 at 14-weeks (p=0.5, 5.7% lower) after surgery (**Table 2**). 2-weeks after surgery CMRO_2_ was significantly higher in the BSC group compared to the HC group (p=0.004) (**Table 2**). 14-weeks following surgery, CMRO_2_ was similar to HC (p=0.17). (**Figure 2**). CBF and OEF were not significantly different between the HC and BSC pre-surgery. CBF and OEF were also not significantly different post versus pre surgery in the BSC group. Please refer to **Table 2** for global CMRO_2_ and the associated global CBF and OEF in HC and BSC groups.

**Table 2:** Global CMRO_2_ and the associated global CBF and OEF in HC and BSC groups.

	Healthy normal weight control (HC) group	Bariatric surgery candidates (BSC) group pre-surgery	Bariatric surgery candidates (BSC) group 2-weeks post-surgery	Bariatric surgery candidates (BSC) group 14-weeks post-surgery
**CMRO_2_ (** *μ* **mol/100g/min)**	177.9 ± 33.8	230.4 ± 32.9 (p=0.02)	234.3 ± 16.2 (p=0.004)	217.3 ± 50.4
**CBF (ml/100g/min)**	66.7 ± 13.4	80.1 ± 10.6	77.9 ± 9.5	68.6 ± 11.5
**OEF (%)**	31.8 ± 5.8	35.2 ± 4.3	37.0 ± 4.3	38.4 ± 5.2

Values are mean ± standard deviation (p-value comparison with HC).

non-significant p-values are not listed in the table.

### Relationship of CMRO_2_ and CBF with obesity

**Figure 3:**
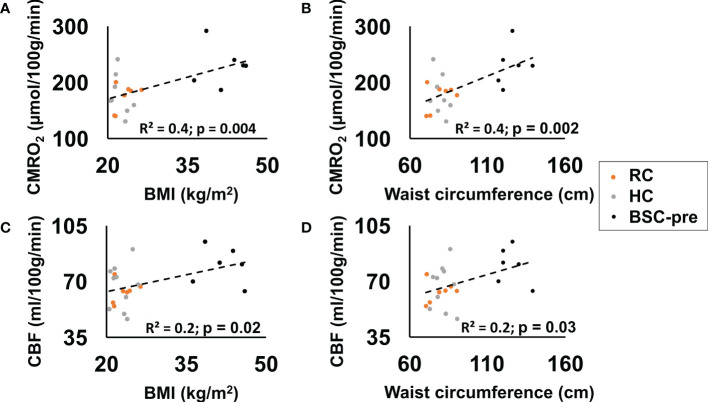
CMRO2 (p=0.004) **(A)** and CBF (p=0.02) **(C)** correlated significantly with BMI (reference control - RC, healthy normal weight controls - HC, bariatric surgery candidates at the pre-surgery stage - BSC-pre). CMRO2 (p=0.002) **(B)** and CBF (p=0.03) **(D)** correlated significantly with waist 7circumference.

CMRO_2_ (p=0.004) and CBF (p=0.02) were significantly correlated with BMI when combining all participants (RC, HC, BSC at the pre-surgery stage) (**Figures 3A, C**). We also observed a significant positive correlation between waist circumference and CMRO_2_ (p=0.002) and CBF (p=0.03) (**Figures 3B, D**).

## Discussion

This exploratory pilot study evaluated the impact of weight loss following sleeve gastrectomy bariatric surgery on cognitive function and CMRO_2_ in BSC with severe/class 3 obesity. Reduction in BMI following bariatric surgery was associated with an increase in cognitive function, aligning with a previous report that found that bariatric surgery was associated with improved neurocognitive function for up to 3 years post-surgery (22, 32).

CMRO_2_ was higher in BSC pre-surgery, compared to HC of a similar age. 2-weeks post-surgery, CMRO_2_ was still higher compared to the HC suggesting that changes do not occur early in the post-operative course, independent of weight loss, probably due to incomplete recovery from surgery requiring more systemic resources. 14-weeks post-surgery, CMRO_2_ in the BSC group was not significantly different compared to the HC group, suggesting that weight loss due to bariatric surgery may be associated with normalization of CMRO_2_ in BSC.

The exact mechanism leading to improved cognitive function post-surgery is not yet understood, but previous research suggests that reduced inflammation, improved glycemic control, reduction in serum leptin level and increase in serum ghrelin level post bariatric surgery may be associated with better cognitive function (33).

Post-surgery diet may also have some effect on CMRO_2_, which cannot be controlled for in these data. The improvement in CMRO_2_ may be associated with the typical lower calorie, low-carbohydrate, liquid diet consumed post-surgery. Post-surgery gastric volume is also smaller, leading to early satiation and a subdued response in the brain to food cues once satiated (34), limiting excess food intake (35) that may lead to less energy supply and demand in the brain and thus potentially to a decrease in CMRO_2_.

We interpret the higher CMRO_2_ in the BSC at baseline to reflect inefficient brain oxygen metabolism. Indeed, research from our group suggests that CMRO_2_ increases with age at about 2.6 µmol O_2_/100 g/min per decade, showing a relationship between increased CMRO_2_ and increasing age, which suggests that increased age and CMRO_2_ are linked to decreased brain function and efficiency (31). We observed a comparable per decade increase with age in CMRO_2_ between the RC and HC group, in line with results from the previous report (31). CMRO_2_ in our RC group is similar to values previously reported (31). Cognitive function decreases with age, while CMRO_2_ increases, possibly due to imbalances in oxygen delivery, consumption, and demand (36). Higher CMRO_2_ in the BSC group supports this hypothesis. This may suggest that obesity accelerates brain ageing. Collectively, our results may suggest that normalization in CMRO_2_ following bariatric surgery may be associated with improvement in overall cognitive function.

The effect of co-morbidities or medication use in the group with obesity cannot be ruled out from this pilot exploratory study. Future studies must focus on the role of diabetes or other metabolic co-morbidities on CMRO_2_ and cognitive function. Studies must also aim to differentiate the effects on these outcomes in participants with obesity and metabolic co-morbidities compared to participants with obesity, without metabolic co-morbidities, to better understand the impact of obesity on health. The impact of different surgical techniques, non-surgical lifestyle/dietary weight loss, anti-obesity medications, and exercise should also be explored in future studies.

### Limitations and future studies

The findings from this exploratory pilot study should be considered with respect to the study limitation that the sample size is small. The results are therefore hypothesis generating. Practice effects cannot be fully ruled out from repeated assessments at baseline and 2-weeks after surgery in participants with obesity. Also, cognitive assessments in the HC group were performed only one time, at baseline, and not repeated the same number of times as in the BSC group.

## Conclusion

In this exploratory pilot study, we evaluated the changes in cognitive function and CMRO_2_ in people with severe obesity who underwent bariatric surgery. The results are hypothesis generating, suggesting that obesity may be associated with metabolic inefficiency and sleeve gastrectomy induced weight loss may improve metabolic efficiency, contributing to improvements in cognitive function.

## Data availability statement

The original contributions presented in the study are included in the article/Supplementary Material. Further inquiries can be directed to the corresponding author.

## Ethics statement

The studies involving human participants were reviewed and approved by Institutional Review Board. The patients/participants provided their written informed consent to participate in this study.

## Author contributions

NA and BT contributed to the data analysis and interpretation of the results, and to the drafting of the manuscript. WT, TS, EG, MN, and BT performed the MRI experiments. WT, TS, EG, and MN performed the cognitive function testing. NP VZ, IL, JA, RB, MN, and BT contributed to the concept and design of the study. JA help with recruiting participants for the study. NP performed the bariatric surgery procedures. JW contributed to the statistical analysis. MN obtained funding for the study. MN and BT supervised the study. All authors edited and approved the final version of the manuscript.
